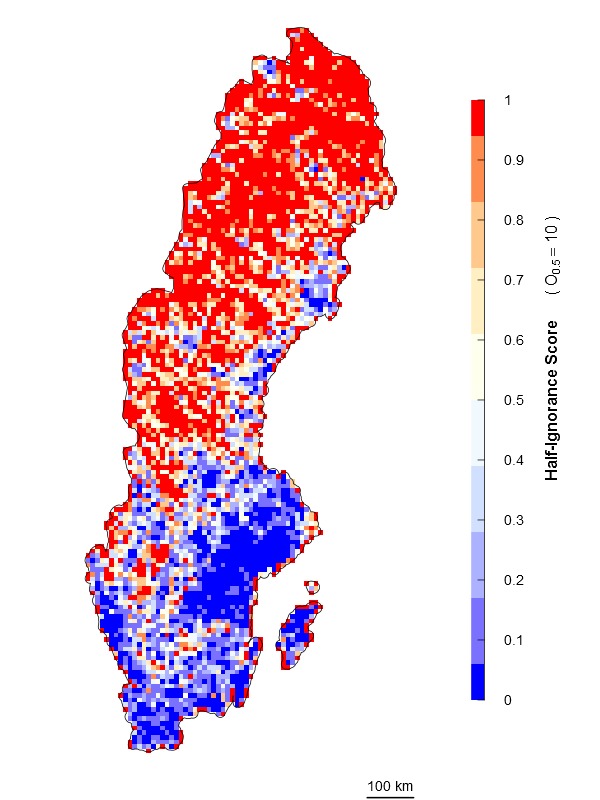# Displaying bias in sampling effort of data accessed from biodiversity databases using ignorance maps

**DOI:** 10.3897/BDJ.3.e5361

**Published:** 2015-07-28

**Authors:** Alejandro Ruete

**Affiliations:** ‡ Swedish University of Agricultural Sciences, Uppsala, Sweden

**Keywords:** Biodiversity database, citizen-science data, presence-only data, sampling effort, spatial bias, species distribution model, Swedish Lifewatch

## Abstract

**Background:**

Open-access biodiversity databases including mainly citizen science data make temporally and spatially extensive species’ observation data available to a wide range of users. Such data have limitations however, which include: sampling bias in favour of recorder distribution, lack of survey effort assessment, and lack of coverage of the distribution of all organisms. These limitations are not always recorded, while any technical assessment or scientific research based on such data should include an evaluation of the uncertainty of its source data and researchers should acknowledge this information in their analysis. The here proposed maps of ignorance are a critical and easy way to implement a tool to not only visually explore the quality of the data, but also to filter out unreliable results.

**New information:**

I present simple algorithms to display ignorance maps as a tool to report the spatial distribution of the bias and lack of sampling effort across a study region. Ignorance scores are expressed solely based on raw data in order to rely on the fewest assumptions possible. Therefore there is no prediction or estimation involved. The rationale is based on the assumption that it is appropriate to use species groups as a surrogate for sampling effort because it is likely that an entire group of species observed by similar methods will share similar bias. Simple algorithms are then used to transform raw data into ignorance scores scaled 0-1 that are easily comparable and scalable. Because of the need to perform calculations over big datasets, simplicity is crucial for web-based implementations on infrastructures for biodiversity information.

With these algorithms, any infrastructure for biodiversity information can offer a quality report of the observations accessed through them. Users can specify a reference taxonomic group and a time frame according to the research question. The potential of this tool lies in the simplicity of its algorithms and in the lack of assumptions made about the bias distribution, giving the user the freedom to tailor analyses to their specific needs.

## Introduction

“The greatest enemy of knowledge is not ignorance; it is the illusion of knowledge.” Daniel J. Boorstin

The emergence of open-access databases on diverse kinds of environmental data (e.g. www.worldclim.org; www.climond.org) and species occurrences data (e.g. www.gbif.org) has led to a rapid increase in biogeographical studies developing new theories, methodologies and applications for nature conservancy (Elith et al. 2010, Franklin 2010, Franklin 2013, Peterson and Soberón 2012). Accurate mapping of species distributions is a fundamental goal of modern biogeography, both for basic and applied purposes. Common mapping techniques are expert-drawn range maps, the plotting of known species occurrences in atlas maps, and geographical estimations derived from species distribution models. However, all three kinds of maps are implicitly subject to uncertainty, due to the quality and bias of raw distributional data, the process of map building, and the dynamic nature of species distributions themselves (Rocchini et al. 2011).

For most species, raw distributional data accessible in biodiversity databases are presence data coming from museums, herbaria, inventories, or citizen science programs, and are the result of a vast number of observers collecting data over a large time span with no specific sampling design (Suarez and Tsutsui 2004). Therefore, biodiversity databases have limitations which include: (1) inadequacy of raw data to describe distribution patterns due to sampling bias in favor of recorder, rather than species distribution (Prendergast et al. 1993), (2) lack of survey effort assessment (Hill 2012), and (3) lack of coverage of the geographic and environmental variations that affect the distribution of organisms (Hortal et al. 2007). Because of these limitations, the results of different mapping techniques differ from the true distribution of the species (Hortal et al. 2007, Schulman et al. 2007). For example, range maps represent actual distributional patterns only at some relatively coarse and undefined resolution, because a species does not occur at all locations within its geographic range (Hurlbert and Jetz 2007). Conversely, most species have not been recorded in some of the grid cells that they actually occupy, and many grid cells have been insufficiently sampled, so atlas maps for almost all regions and taxa present broad geographical gaps in knowledge (Hurlbert and Jetz 2007). Finally, spatial bias in the records may translate into a biased relationship between species occurrence and environmental variables (Hertzog et al. 2014). Presence-only datasets require special treatment and assumptions before use, because uncorrected models show a strong bias in their predicted patterns (Hertzog et al. 2014). As a consequence, a method for quantifying how much recording effort a given location has received based upon presence-only observation records is required.

All these issues stemming from the quality of the raw data can be ameliorated by the use of parallel “maps of ignorance” to provide information on sampling coverage and reliability (Hortal et al. 2007, Rocchini et al. 2011). Good practice in science requires the assessment, statement, and acknowledgement of measurement error: any technical assessment, monitoring program, or scientific research should thus include an evaluation of the uncertainty of its results. Therefore, publishers of open-access databases should inform about the data quality, as researchers should acknowledge this information in their analysis. However, such quality control is rarely available to users of biodiversity databases (Hortal 2008).

I present simple algorithms to create and display ignorance maps based upon presence-only observation records. The algorithms are thought to be general enough to be implemented as web-based tools to download ignorance scores in the form of raster images. Ignorance maps will serve to properly inform users of the bias inherent to the data and to provide them with tools to properly analyse the raw data provided. The approach presented here is in line with the need identified by Rocchini et al. (2011) and will provide quality control tools for protocols for biodiversity analysis such as the one proposed by Hortal et al. (2007). In this article I describe the algorithms and considerations needed to produce these ignorance maps, as well as examples of their potential uses, so that they could be implemented either by biodiversity databases or directly by researchers. Particularly, these algorithms are currently being implemented by The Swedish LifeWatch (SLW, www.svenskalifewatch.se), a national e-infrastructure for integration and analysis of biodiversity data (Gärdenfors et al. 2014) that assembles mainly presence-only non-systematic observations. The performance of the algorithms applied to real Swedish data can be explored using an HTML application run through R that can be downloaded from the project website (http://alejandroruete.github.io/IgnoranceMaps) and the code is available to be adapted to other study cases.

## Project description

### Title

Ignorance maps of raw data accessed from species observation databases

### Study area description

Worldwide; example data from Sweden​

### Design description

**Rationale and assumptions**

The aim is to provide ignorance maps that are easily comparable and easily scalable, to report the spatial distribution of sampling effort (or lack of it). Therefore the obvious choice is to represent ignorance on a scale of 0 to 1 (1 being absolute ignorance and 0 being absolute certainty or credibility in the data). There are several approaches to incorporate sampling effort to different analysis of richness, species distributions and trends in population abundance (Hill 2012, Jeppsson et al. 2010, Ponder et al. 2001, Prendergast et al. 1993, Schulman et al. 2007, Snäll et al. 2011). However, most of these methods require several assumptions that constrain their generality and comparability. Conversely, the aim of this approach is to express ignorance solely based on raw data summarized per grid cell in order to rely on the fewest assumptions possible. The aim is not to include any covariates or correlation and to avoid prediction, estimation and interpolation methods (see e.g. Ponder et al. 2001). These basic criteria will give the end-user more freedom to adapt the ignorance maps to their own research question.

Observations are reported by people with varied field skills and accuracy. Because of the intrinsic characteristics of the reports (e.g. voluntary, non-systematic), biodiversity datasets have a considerable spatial and temporal bias. However, observers are assumed to be fond of or specialist on one or more taxonomic groups (e.g. family, order), rather than on individual species. Since it is likely that an entire group of species observed by similar methods (henceforth a reference taxonomic group) will share similar bias (Phillips et al. 2009), it is appropriate to use species’ groups as a surrogate for sampling effort (Phillips et al. 2009, Ponder et al. 2001). Therefore, it is straightforward to assume that the lack of reports of any species from the reference taxonomic group (e.g. birds) at a particular location is likely due to a lack of ornithologists on that specific location, rather than to the total absence of birds. The inverse logic also holds true. That is, the larger the number of observations of species from the reference taxonomic group in a grid cell, the more likely it is that the lack of reports of a particular species reflects a true absence of that species from the grid cell (i.e. larger certainty).

There are some considerations to take into account before describing the algorithms. First, the reference target group should only include species that are assumed to be sampled with the same methodology, to keep the sampling bias consistent (Ponder et al. 2001). For example, reference taxonomic groups should not include all species in the Order Lepidoptera because butterflies *sensu stricto* (superfamily Papilionoidea) are sampled in very different ways than all other species of Lepidoptera (mainly moths). Alternatively, interacting species could be included for specialist and symbiont species. Second, it has been pointed out that in case that ignorance maps are to be used to correct the sampling bias of background information (for software packages like MaxEnt; http://www.cs.princeton.edu/~schapire/maxent/), the target species should be removed from the reference taxonomic group if it is known that the species has been heavily sampled at a particular location but has few records in the vicinity (Ponder et al. 2001). In the case of allopatric species, however, removing the target species will leave “holes” in the ignorance maps (Ponder et al. 2001). Finally, it is preferred to calculate ignorance maps including observations over long time periods to reduce temporal variability in sampling effort (Snäll et al. 2011). Of course, this is only valid as long as there is no significant change to the underlying habitat that holds the species, and time itself is not a covariate to be included in the analysis of the data.

**Algorithms overview**

The sampling behaviour that characterizes observers differs among reference taxonomic groups. For some groups like vascular plants or bryophytes observers typically inventory confined areas (sites) reporting every species they observe, aiming to cover as many sites as possible. In these cases, raw observation counts per grid cell *i* (*N_i_*) better represent the sampling intensity and species discovery (Fig. 1a). For other groups like birds, observers aim to complete a species list and often have preferred observation sites. Also, common species within these groups are often not reported by voluntary citizen scientists (Snäll et al. 2011). In these cases, a species observation index \begin{varwidth}{50in}
        \begin{equation*}
            O{i} = \left\{\begin{array}{l l} 0, & N_{i} = 0\\ N_{i}/R_{i}, & N_{i} > 0 \end{array} \right.;
        \end{equation*}
    \end{varwidth} is preferred, where *R_i_* is the number of species observed in grid cell *i.* The species observation index *O_i_* offsets the sampling effort relative to the number of species reported per grid cell. The relationship between the number of observations and the species observation index is shown in Fig. 1b for different reference taxonomic groups including mammals (land mammals without bats), birds, butterflies (superfamily Papilionoidea) and vascular plants (Tracheophyta). The use of *N_i_* or *O_i_* is optional to the researcher, and its consequences can be further explored using the HTML application run through R that can be downloaded from the projects webpage (http://alejandroruete.github.io/IgnoranceMaps). For simplicity, in this article “number of observations” will also refer to the species observation index.

**Figure 2.**
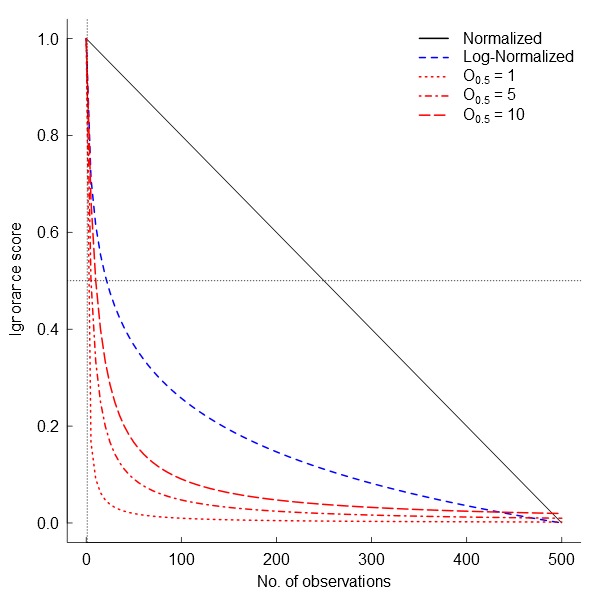
Ignorance scores as a function of the number of observations per grid cell. The curves for the half-ignorance algorithm (red lines) are calculated for three values of *O*_0.5_ = 1, 5, and 10 (i.e. enough number of observations to reduce the ignorance score by half).

The first and easiest way to transform observation counts into a 0-1 scale of ignorance (I) is by using normalized data (henceforth the Normalization approach): \begin{varwidth}{50in}
        \begin{equation*}
            I_{i}=1-N_{i}/N_{m}
        \end{equation*}
    \end{varwidth} where *N_m_* is the maximum number of observations per grid cell of the dataset. Then 0 represents the maximum certainty of the data corresponding to the maximum number of observations recorded in the entire dataset (Fig. 2) and 1 represents absolute ignorance. The normalization algorithm is recommended when the maximum number of observations is not too different from the mean number of observations, and particularly for areas with low variability. However, it is not recommended when the probability distribution of number of observations per grid cells presents a long right tail (i.e. many grid cells with none or few observations and few cells with extremely high number of observations).

In many cases there are sites that are more than sufficiently sampled (i.e. long right tails in the probability distribution of observations) but the relative influence of these sites on our certainty may not be linear. In these cases, when it is relevant to separate sites with “few” observations from sites with “enough” observations, logarithmic transformations are preferred (Fig. 2). Then, ignorance is equal to one minus the normalization of the natural logarithm of the data (henceforth the Log-Normalization approach) \begin{varwidth}{50in}
        \begin{equation*}
            I_{i}=1-\log(N_{i}+1) / \log(N_{m}+1)
        \end{equation*}
    \end{varwidth}. A unit is added before log-transforming the data so that grid cells without observations are transformed to the highest ignorance score, i.e. 1. In both algorithms presented so far the minimum ignorance score, i.e. 0, is relative to the maximum number of observations for the reference taxonomic group. Therefore, ignorance maps produced with these algorithms are highly sensitive to the spatial and temporal extent of the data because the absolute maximum may not be included in subsets of the dataset.

An alternative approach is an algorithm independent of the maximum number of observations. It estimates ignorance scores making data relative to a reference number of observations that is considered to be enough to reduce the ignorance score by half (henceforth the Half-ignorance approach). In this case, ignorance scores are defined as \begin{varwidth}{50in}
        \begin{equation*}
            I_{i} = O_{0.5}/(N_{i}+O_{0.5})
        \end{equation*}
    \end{varwidth} (Fig. 2). In other words, setting the reference number *O*_0.5_ = 1 means that one observation is enough to consider that the absence of reports of a target species from any grid cell is 50% due to true absence from the site and 50% due to failure to detect the species. Setting *O*_0.5_ < 1 denotes more confidence on every single observation, not gaining much information from a higher number of observations. In this case, setting *O*_0.5_ = 0.5 assumes that the first single observation (of any species in the reference taxonomic group) reduces our ignorance to 0.333. Conversely, setting *O*_0.5_ > 1 denotes the need for more than one observation per grid cell to rely on such information (i.e. to significantly reduce the ignorance score). For example, setting *O*_0.5_ = 5 assumes that we need at least five observations to partially trust on the sampling effort spent in any particular grid cell. This algorithm allows the researcher to customize its credibility on each observation in a way that the ignorance score approaches asymptotically to 0 as the number of observation increases. However, the bigger the *O*_0.5_ the slower ignorance scores will approach 0 (Fig. 2). This approach is specially recommended when i) there are particular assumption about the confidence on each observation, and ii) when the aim is to compare datasets with very different maximum number of observaitons. As an illustration raw observational data for the superfamily Papilionoidea (Fig. 3a) is compared to ignorance maps produced with the three different algorithms: Normalization (Fig. 3b), Log-Normalization (Fig. 3c) and Half-Ignorance algorithms, setting *O*_0.5 _= 1, 5, and 10 (Fig. 3d, e, f respectively). For more examples on different reference taxonomic groups download and run the interactive examples available in http://alejandroruete.github.io/IgnoranceMaps.

It is important to highlight that the size of the grid cells (i.e. resolution) will affect the results of all implemented algorithms. For example, consider the simple case where one large grid cell is made up of four smaller cells of which three cells are empty and only one cell scores all the reported observations. In this case the spatial distribution of recording effort will look very different when mapped at a high or low resolution. Sensitivity to spatial resolution is a common problem on studies summarizing biodiversity data on arbitrary grid cells, and the relevance of this problem has to be evaluated for each study in light of the question or hypothesis tested (Hurlbert and Jetz 2007). The algorithms allow the user to specify the temporal and spatial extent and resolution in order to produce ignorance maps that are relevant to the species biology and researchers needs (note: to do so with the R scripts provided on the project website, the user has to simply replace the raster images with the number of observations and the number of species with the desired ones). Some solutions have been developed to produce scale and resolution independent maps of the sampling effort (i.e. the opposite of ignorance; Schulman et al. 2007). However, because the algorithms suggested by Schulman et al. 2007 are based on Thiessen polygons and interpolations computed for individual observation points (i.e. instead of summaries per grid cells) these solutions are not flexible enough and are too computationally intensive to provide custom web-based results over large datasets.

### Funding

This project was framed within and funded by the Swedish LifeWatch.

## Web location (URIs)

Homepage: www.swedishlifewatch.se

Download page: http://alejandroruete.github.io/IgnoranceMaps

## Technical specification

Platform: ANY

Programming language: ANY. Examples provided as an HTML application programmed in R.

Operational system: ANY

Interface language: English

## Repository

Type: Git

Location: https://github.com/AlejandroRuete/IgnoranceMaps

## Usage rights

### Use license

Other

### IP rights notes

GNU GPL 3.0

DOI: 10.5281/zenodo.17593

## Implementation

### Implements specification

The code provided in the repository is implemented as an HTML application with a local R server through the package *shiny*. The core algorithms introduced here are not dependent on any language and can be used independently or be implemented on biodiversity data portals. For example, the Swedish Lifewatch analysis portal is currently implementing this algorithms in the JAVA language.

The R code provided is adapted to run under the *shiny* server framework, however those who need can find the core algorithms in plain R language in the file "SLWApp/server.r" provided in the repository. This R code and examples will remain in the repository for individual implementations and modifications.

In order to use the R code as is with other species the requirements are:

1. a raster image where each pixel summarizes the total number of observations recorded for the reference taxonomic group during the desired time frame
2. a raster image where each pixel summarizes the total number of individual species within the reference taxonomic group observed during the desired time frame
3. a raster image where each pixel summarizes the total number of observations recorded for the focal species during the desired time frame
4. (Optional) a shape file (.shp) with the contour of the study region

Note that all raster images must have the exact same extent and resolution. In the examples presented here these raster images were created transforming the grid-based summary tables obtained from the Swedish LifeWatch analysis portal into. tiff georeferenced images. Some portals (e.g. GBIF) will only be able to download individual observation data points, in which case the user will need to summarize the data into raster images.

Note as well that although this code is implemented to calculate ignorance scores per pixel, the algorithms can be applied to summaries of irregular areas.

### Audience

Database users can assess, with three alternative algorithms, the spatial bias of the sampling effort and relative amount of knowledge gained for any reference taxonomic group, and download these mapped ignorance data as GIS-layers. End-users will be able to individually set the scale, resolution, time frame, and reference taxonomic groups of interest to assess the utility of the observations reported in the database. Potential target users of the ignorance maps are: 1) consultants performing environmental impact assessments (e.g. they could use ignorance maps to make precautionary statements about lack of knowledge about species of special conservation interest on areas where projects are intended to be developed); 2) observers (e.g. they might be interested in locating under-sampled areas to be targeted on their next campaign); and 3) researchers (they might benefit in many different ways, some of which we describe below).

The most obvious use for ignorance maps is to mask out from other raster layers derived from the raw data (e.g. estimates of pseudo-absence or population abundance) areas of high uncertainty, excluding them from further analyses. A user-defined ignorance threshold could be used to generate pseudo-absences on sites where focal species are likely to be absent given the species has not been observed and that the site counts with high sampling effort for the reference taxonomic group (Hertzog et al. 2014). Conversely, high ignorance scores can identify under-sampled areas where the absence of species observations are less likely to be due to true species absences. In this way, multiplying the opposite of the ignorance map (1− ignorance = certainty) by any other map of occurrence or abundance estimates for focal species will weight these later estimates to the knowledge available (see examples of pseudo-absences estimates multiplied by ignorance maps in the interactive application available in http://alejandroruete.github.io/IgnoranceMaps). Even more, ignorance layers can correct the bias present in comparisons of species composition (Barnes et al. 2014), allowing for more accurate assessment of species richness.

Ignorance maps are of particular interest for species distribution modelling (SDM), as estimates can be improved by incorporating information on how recording effort varies spatially (Stolar and Nielsen 2015). Major improvements in the goodness of fit of machine learning species distribution models (e.g. MaxEnt) can be achieved by directly incorporating ignorance maps as confidence or bias layers for background sampling (Phillips et al. 2009, Syfert et al. 2013). Presence only data from non-systematic sampling effort may be biased by geographical variables, such as altitude or road density, that may also be correlated to each other. Therefore, it may be more informative to use a spatial bias layer such as an ignorance map, and incorporate this layer into the model as an explanatory variable than trying to identify which geographical variable is explaining the bias. In this way the model is explicitly accounting for uncertainty, which can improve model predictions (Stolar and Nielsen 2015).

Within the Bayesian framework SDMs could also benefit by using ignorance scores to inform *a priori* probability distributions (Argáez et al. 2005, McCarthy and Masters 2005). For example, *a priori* probabilities of occurrence of a species for unobserved sites could be generated assuming that occurrences follow a Bernoulli distribution with \begin{varwidth}{50in}
        \begin{equation*}
            p_{i}=1-(1-I_{i} + (0.5\cdot I_{i}))
        \end{equation*}
    \end{varwidth}. Then, for each estimation iteration, an unobserved site with high ignorance, i.e. *I_i_* = 1, could take the value 0 or 1 with the same probability; while an unobserved site with low ignorance score will most likely take the value 0. Then, maps produced from such SDMs can indicate which areas of the study region are most affected by under-sampling and therefore have the greatest predictive uncertainty.

## Additional information

**​Conclusion**

Dealing with uncertainty in presence-only citizen science data is necessary for a wide range of applications, and the development of an ignorance score as implemented here provides an appropriate scale to compare different taxa, and a straight forward and easily interpretable method of doing so. Any infrastructure for biodiversity information on virtually any web infrastructure can offer a quality report of the spatial bias of observations stored in databases implementing these simple algorithms. Quantifying recording effort in citizen science biodiversity datasets allows users to incorporate uncertainty into analyses of species’ richness and distributions, to identify unreliable analyses results, and to identify areas where further surveys are required. Users can specify a reference taxonomic group and a time frame according to the research question. The potential of this tool lies in the simplicity of its algorithms and the lack of assumptions made about the bias distribution, giving the user the freedom to tailor analyses to their specific needs.

## Supplementary Material

Supplementary material 1 Number of observations and number of species per grid cell (.CSV) Data type: Summary of occurrences and richness per grid cell (.CSV)
Brief description: The algorithms are designed to handle number of observations and number of species summarized per grid cells. Here I provide the. CSV files as downloaded from www.analysisportal.se. This is the format one is expected to get the summarized data for a biodiversity database. I also include data on the occurrence of two species (a common and a rare) for each reference taxonomic group.
File: oo_43444.zip Swedish LifeWatch / Swedish Species Infromation Centre

Supplementary material 2 Number of observations and number of species per grid cell (.TIFF) Data type: Summary of occurrences and richness per grid cell (.TIFF)
Brief description: The algorithms are designed to handle number of observations and number of species summarized per grid cells. Here I provide the raster images used for the examples provided in the R script available on the GitHUB repository. These images were produced from. CSV files downloaded from www.analysisportal.se
Amp=Amphibians; MamLnB=Land Mammals; Bir=Birds; Odo=Odonata; Opi=Opilionidae; Pae=Papilionoidea; Vas=Vascular Plants
File: oo_43439.zip Swedish LifeWatch / Swedish Species Information Centre

## Figures and Tables

**Figure 1a.**
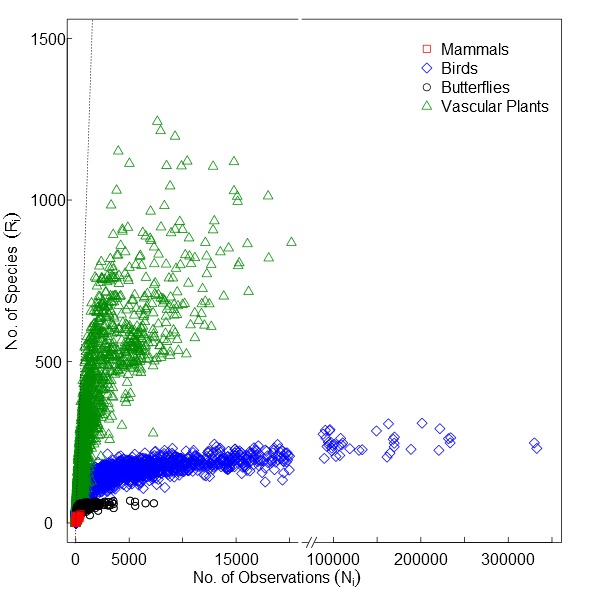


**Figure 1b.**
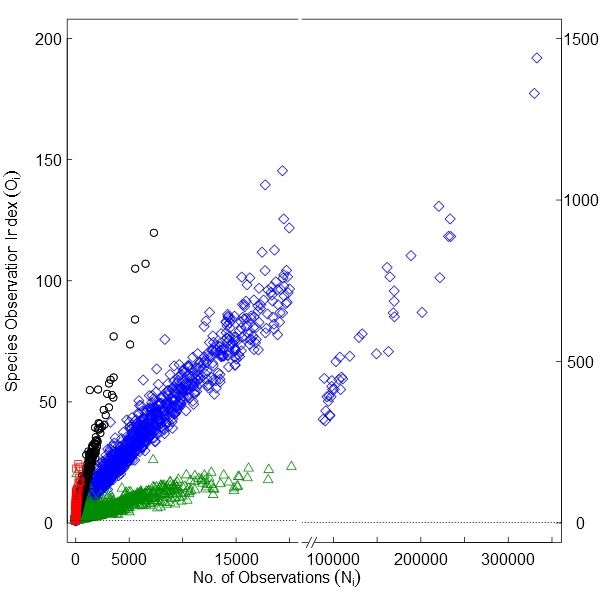


**Figure 3a.**
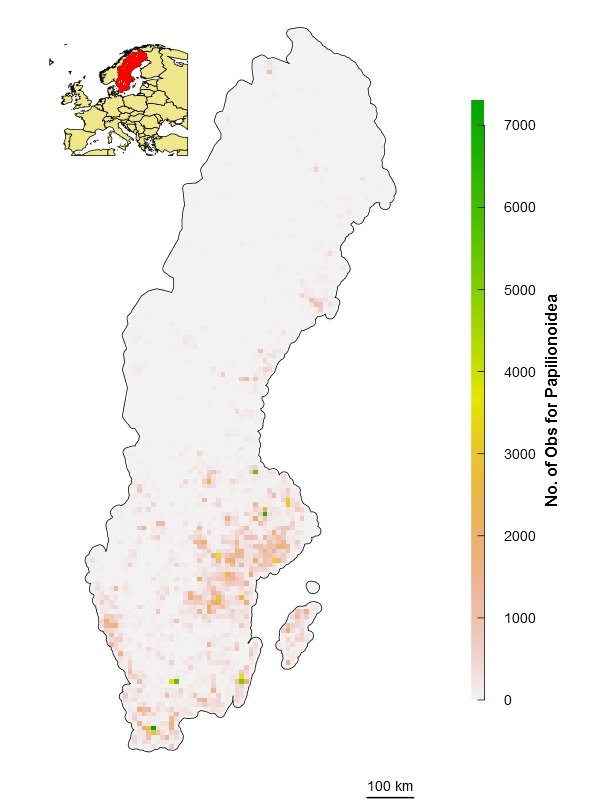


**Figure 3b.**
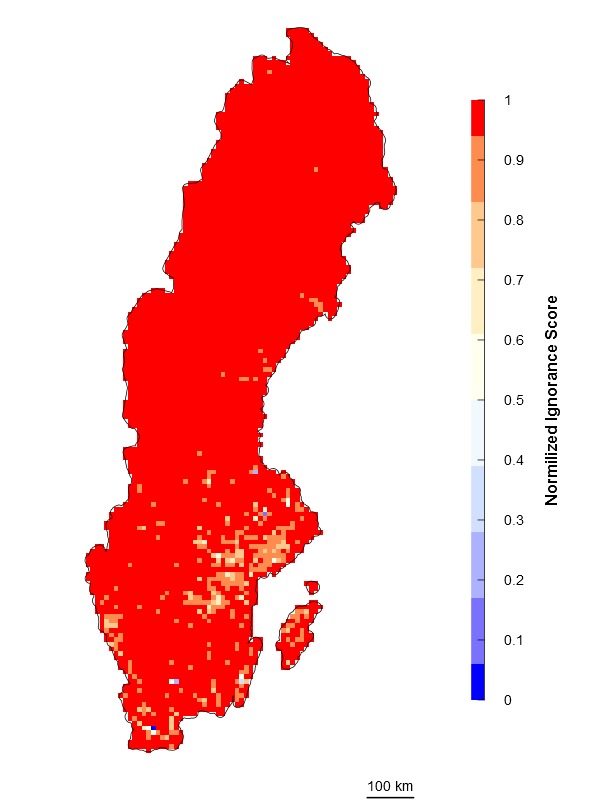


**Figure 3c.**
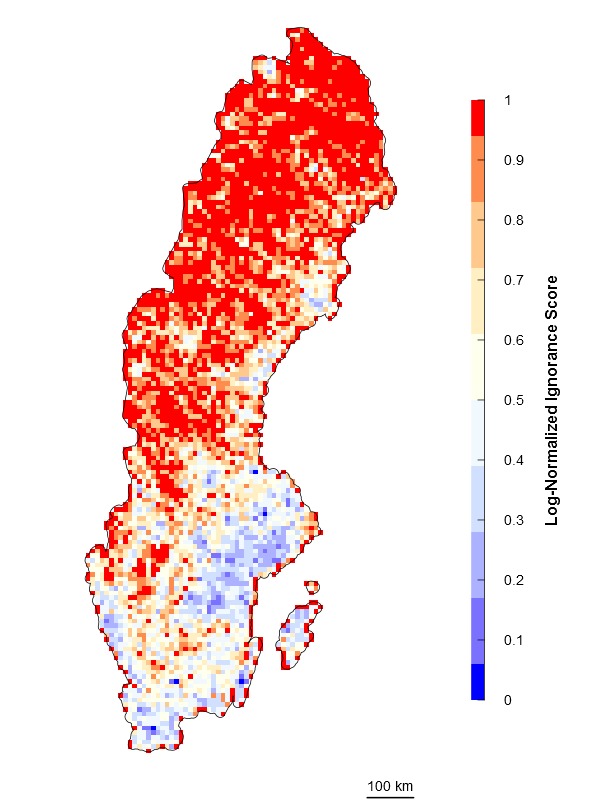


**Figure 3d.**
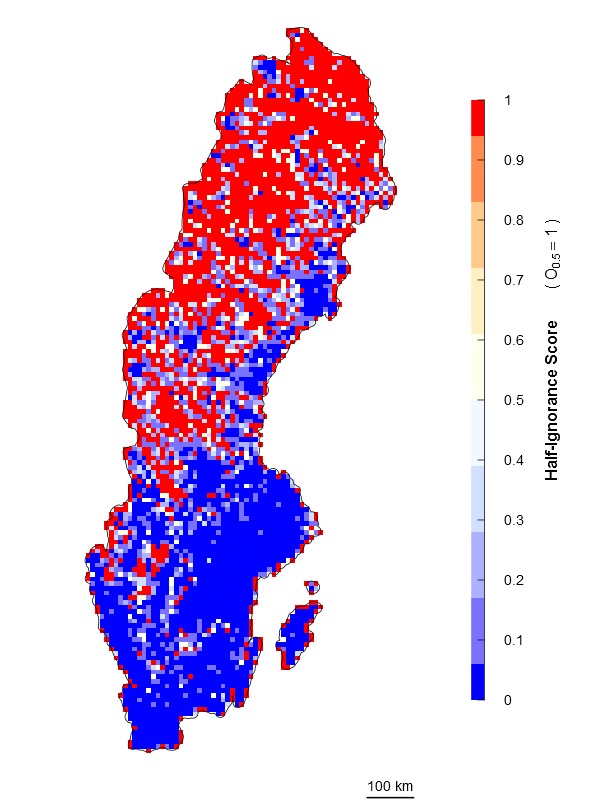


**Figure 3e.**
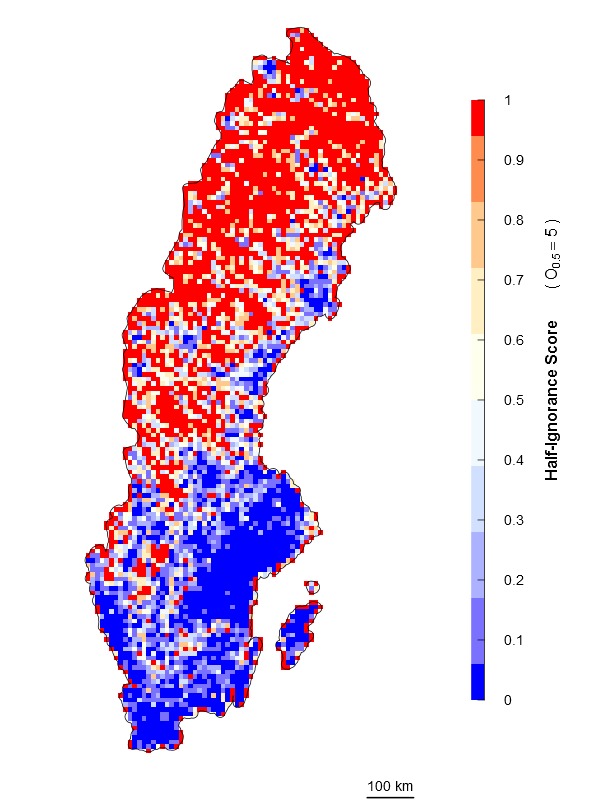


**Figure 3f.**